# A Comparative Study of Two-Compartment Exchange Models for Dynamic Contrast-Enhanced MRI in Characterizing Uterine Cervical Carcinoma

**DOI:** 10.1155/2019/3168416

**Published:** 2019-11-07

**Authors:** Xue Wang, Wenxiao Lin, Yiting Mao, Wenwen Peng, Jiao Song, Yi Lu, Yu Zhao, Tong San Koh, Zujun Hou, Zhihan Yan

**Affiliations:** ^1^Department of Radiology, The Second Affiliated Hospital and Yuying Children's Hospital of Wenzhou Medical University, 109 Xueyuanxi Road, 325027 Wenzhou, China; ^2^Wenzhou Medical University, Gaojiaoyuan District, 325027 Wenzhou, China; ^3^Department of Gynaecology and Obstetrics, The Second Affiliated Hospital and Yuying Children's Hospital of Wenzhou Medical University, 109 Xueyuanxi Road, 325027 Wenzhou, China; ^4^Department of Oncologic Imaging, National Cancer Center 169610, Singapore; ^5^Duke-NUS Graduate Medical School 169547, Singapore; ^6^Suzhou Institute of Biomedical Engineering and Technology, Chinese Academy of Sciences, Suzhou 25163, China

## Abstract

A variety of tracer kinetic methods have been employed to assess tumor angiogenesis. The Standard two-Compartment model (SC) used in cervix carcinoma was less frequent, and Adiabatic Approximation to the Tissue Homogeneity (AATH) and Distributed Parameter (DP) model are lacking. This study compares two-compartment exchange models (2CXM) (AATH, SC, and DP) for determining dynamic contrast-enhanced magnetic resonance imaging (DCE-MRI) parameters in cervical cancer, with the aim of investigating the potential of various parameters derived from 2CXM for tumor diagnosis and exploring the possible relationship between these parameters in patients with cervix cancer. Parameters (tissue blood flow, *F*_p_; tissue blood volume, *V*_p_; interstitial volume, *V*_e_; and vascular permeability, PS) for regions of interest (ROI) of cervix lesions and normal cervix tissue were estimated by AATH, SC, and DP models in 36 patients with cervix cancer and 17 healthy subjects. All parameters showed significant differences between lesions and normal tissue with a *P* value less than 0.05, except for PS from the AATH model, *F*_p_ from the SC model, and *V*_p_ from the DP model. Parameter *V*_e_ from the AATH model had the largest AUC (*r* = 0.85). Parameters *F*_p_ and *V*_p_ from SC and DP models and *V*_e_ and PS from AATH and DP models were highly correlated, respectively, (*r* > 0.8) in cervix lesions. Cervix cancer was found to have a very unusual microcirculation pattern, with over-growth of cancer cells but without evident development of angiogenesis. *V*_e_ has the best performance in identifying cervix cancer. Most physiological parameters derived from AATH, SC, and DP models are linearly correlated in cervix cancer.

## 1. Introduction

Neovascularization plays a fundamental role in the growth of solid tumors [1]. Various experimental models as well as clinicopathological observations have shown that solid tumors (e.g., breast [2], lung [3], and cervical carcinoma [4, 5]) cannot attain diameters >2-3 mm without their own vascular supply. Dynamic contrast-enhanced magnetic resonance imaging (DCE-MRI) is commonly used for the assessment of tumor angiogenesis. Analysis of DCE-MRI data can be performed using tracer kinetic models to derive quantitative parameters of tissue microcirculation. A variety of tracer kinetic methods have been employed to characterize various tumors and to assess the effects of antiangiogenic and antivascular drugs in clinical trials [6–15].

To date, the Generalized Kinetic (GK or Tofts) model and the Extended Generalized Kinetic (EGK or extended Tofts) model are frequently used for analysis of DCE-MRI data in oncology and drug trials. It is commonly assumed that *K* ^trans^ yielded from the two models encompasses the effects of both blood flow and vessel permeability, such that *K* ^trans^ reflects permeability when blood flow is much larger than permeability and *K* ^trans^ reflects blood flow when permeability dominates [16]. However, novel vascular targeting drugs could reduce both permeability and blood flow and might exert different pharmacokinetic effects on blood flow and permeability [17, 18]. Thus, a method that can separately estimate tissue blood (plasma) flow (*F*_p_) and vessel permeability (PS) is more valuable for the assessment of drug effects. Improvements in the temporal resolution of DCE-MRI sequences [19] have facilitated independent measurement of *F*_p_ and PS using the two-compartment exchange model (2CXM). A few such methods have been proposed, which include the Standard two-Compartment model (SC), Adiabatic Approximation to the Tissue Homogeneity (AATH), and the two-compartment Distributed Parameter model (DP) [20–23].

Several studies have applied these models in cervix carcinoma. The relationship between parameters from various tracer kinetic methods [24, 25] has been reported. However, these studies mainly focused on GK and EGK models, and application of the SC model to cervix cancer was less frequent. Kallehauge et al. found that *K* ^trans^ mainly reflects tissue blood flow even though PS < *F*_p_, which is contradictory with the understanding on *K* ^trans^ which will reflect flow when PS is infinite [25]. Donaldson et al. observed that the correlation coefficient of *K* ^trans^ with PS is low (*r* = 0.31), while the correlation coefficient of *K* ^trans^ with *F*_p_ is high (*r* = 0.94) [24]. *V*_e_ yielded from the EGK model was correlated with 2CXM, but correlations between *V*_p_ from EGK and 2CXM were different for different studies [24, 25]. The application of AATH and DP models in cervix carcinoma is still lacking. So, parameter variations assessed by the two-compartment exchange models (AATH, SC, and DP) with respect to tumor microcirculation would be clinically desirable. Moreover, to facilitate the consistent interpretation of treatment effects for multicenter clinical trials employing different tracer kinetic models, it would be of interest to understand the relationships between parameters of these tracer kinetic models, so that if one kinetic model is used in a certain trial, one could get a sense of what would be the likely changes in another kinetic model. In this study, three two-compartment models (AATH, SC, and DP) were applied in cervix carcinoma to investigate the potential of various parameters in tumor diagnosis and to examine the possible relationship between parameters of these tracer kinetic models.

## 2. Materials and Methods

### 2.1. Patients

This retrospective study was approved by the institutional research ethics review board, and informed consent was obtained from all patients. Sixty-eight consecutive female patients (mean age, 50.4 years; age range, 41–75 years) clinically suspected of cervix cancer presented to our department in the period of April 2016 to July 2018. Patients were excluded for the following reasons: (1) poor image quality of DCE-MRI such as significant motion artifacts (*n* = 1) or incomplete images (*n* = 2); (2) patients with a history of targeted chemotherapy or radiation therapy before examination (*n* = 4); (3) patients diagnosed of other cervical lesions, such as submucous myoma of uterus (*n* = 2) and the endometrial carcinoma (*n* = 1); and (4) no mass was identified for patients with stage Ia on DCE and other MRI sequences (*n* = 5). In the end, 36 patients with cervix cancer and 17 healthy subjects were included in this retrospective study. Cervix cancer was clinically staged according to the International Federation of Gynecology and Obstetrics classifications [26]. These 36 patients were classified into stage Ib (*n* = 17), IIa (*n* = 14), and IIb (*n* = 5). All patients were confirmed histopathologically. Hysterectomy was performed for cervix cancer patients and biopsy for healthy subjects. Patient characteristics are summarized in Table 1.

### 2.2. MR Imaging Protocol

All scans were performed on a 3.0 T scanner (Discovery™ MR750w, General Electric, USA) using an 8-channel torso phased-array coil. Routine clinical MRI scan sequences included a transverse fast spin-echo T1-weighted sequence (repetition time/echo time = 550–700/7–10 ms; field of view = 320 × 320 mm; matrix size = 512 × 512; slice thickness = 5.0 mm; intersection gap = 6 mm), a short time inversion recovery (STIR) T2-weighted sequence (repetition time/echo time = 3000–4000/70–80 ms; field of view = 256–320 × 256–320 mm; matrix size = 512 × 512; slice thickness = 5.0 mm; intersection gap = 6–7 mm), and diffusion-weighted imaging (DWI) (repetition time/echo time = 2400/60 ms, field of view = 320 × 320 mm, matrix size = 256 × 256 mm; slice thickness = 5.0 mm, intersection gap = 7 mm, *b* value = 0, 1000 s/mm^2^).

DCE-MRI was performed using a three-dimensional T1-weighted spoiled gradient echo sequence (LAVA, repetition time/echo time = 3/1 ms, flip angle = 4°, 8° and 11°, field of view = 360 × 360 mm, matrix size 256 × 256, slice thickness = 5 mm, 6 slices per slab). Ten precontrast scans of each flip angle (4°, 8°, and 11°) were acquired in the axial plane under quiet respiration. Dynamic postcontrast scans were acquired using the same sequence and a flip angle of 11°, with the intravenous injection of gadopentetated imeglumine (Magnevist; Bayer Healthcare Pharmaceuticals Inc., NJ) at an injection rate of 2 mL/second standard dose of 0.1 mmol/kg. A total of 180 consecutive scans were acquired for the dynamic series with a temporal resolution 2 s. Subsequently, a routine late contrast-enhanced T1-wieghted scan (repetition time/echo time = 4/2 ms, flip angle = 13°, field of view = 280 × 280 mm, matrix size 512 × 512, slice thickness = 3 mm) was acquired in the sagittal plane.

### 2.3. Tracer Kinetic Models

Details of the four tracer kinetic models used in this study can be found in several review papers [21, 22]. Here, we would only list the essential operational equations for these models which specify the dependence of tissue tracer concentration *C*_tiss_(*t*) (as a function of time *t*) on the arterial input function (AIF) and relevant physiological parameters:

  Standard Compartment (SC) model

(1a) $$\begin{array}{c} C_{\text{tiss}}\left(t\right)=\text{ AIF }⊗\;F_{\text{p}}\left[A\;\exp\left(\alpha \;t\right)+\left(1-A\right)\exp\left(\beta \;t\right)\right], \end{array}$$

  where

(1b) $$\begin{array}{c} \left(\begin{array}{c} \alpha \\ \beta \end{array}\right)=\frac{1}{2}\left[-\left(\frac{\text{PS}}{V_{\text{p}}}+\frac{\text{PS}}{V_{\text{e}}}+\frac{F_{\text{p}}}{V_{\text{p}}}\right)\pm \sqrt{{\left(\frac{\text{PS}}{V_{\text{p}}}+\frac{\text{PS}}{V_{\text{e}}}+\frac{F_{\text{p}}}{V_{\text{p}}}\right)}^{2}-4\frac{\text{PS}}{V_{\text{e}}}\frac{F_{\text{p}}}{V_{\text{p}}}}\right], \end{array}$$

(1c) $$\begin{array}{c} A=\frac{\alpha +\left(\text{PS}/V_{\text{p}}\right)+\left(\text{PS}/V_{\text{e}}\right)}{\alpha -\beta }, \end{array}$$

  and ⊗ denotes the convolution operator.
  Distributed Parameter (DP) model

(2) $$\begin{array}{c} C_{\text{tiss}}\left(t\right)=\text{AIF}⊗,\\ F_{\text{p}}\left\{u\left(t\right)-u\left(t-\frac{V_{\text{p}}}{F_{\text{p}}}\right)+u\left(t-\frac{V_{\text{p}}}{F_{\text{p}}}\right)\left\{\left.1-\exp\left(-\frac{\text{PS}}{F_{\text{p}}}\right)\left[1+\int_{0}^{t-\left(V_{\text{p}}/F_{\text{p}}\right)}\exp\left(-\frac{\text{PS}}{V_{\text{e}}}\tau \right)\sqrt{\frac{\text{PS}}{V_{\text{e}}}\;\frac{\text{PS}}{F_{\text{p}}\;}\frac{1}{\tau }}\;I_{1}\left(2\sqrt{\frac{\text{PS}}{V_{\text{e}}}\;\frac{\text{PS}}{F_{\text{p}}}\tau \;}\right)\text{d}\tau \right]\right.\right\}\right\}, \end{array}$$

  where *u*(*t*) denotes the Heaviside unit-step function and *I*_1_ is the modified Bessel function.
  Adiabatic Approximation to the Tissue Homogeneity (AATH) model

(3) $$\begin{array}{c} C_{\text{tiss}}\left(t\right)=\text{AIF}⊗,\\ F_{\text{p}}\left\{\begin{array}{c} \left[1-\exp\left(-\frac{\text{PS}}{F_{\text{p}}}\right)\right]\exp\left\{-\frac{F_{\text{p}}}{V_{\text{e}}}\left[1-\exp\left(-\frac{\text{PS}}{F_{\text{p}}}\right)\right]\left(t-\frac{F_{\text{p}}}{V_{\text{p}}}\right)\right\} \end{array}\right\}. \end{array}$$

Interested readers can refer to recent review papers [21, 22] for more details of the three tracer kinetic models.

### 2.4. Image Postprocessing

**Figure 1:**
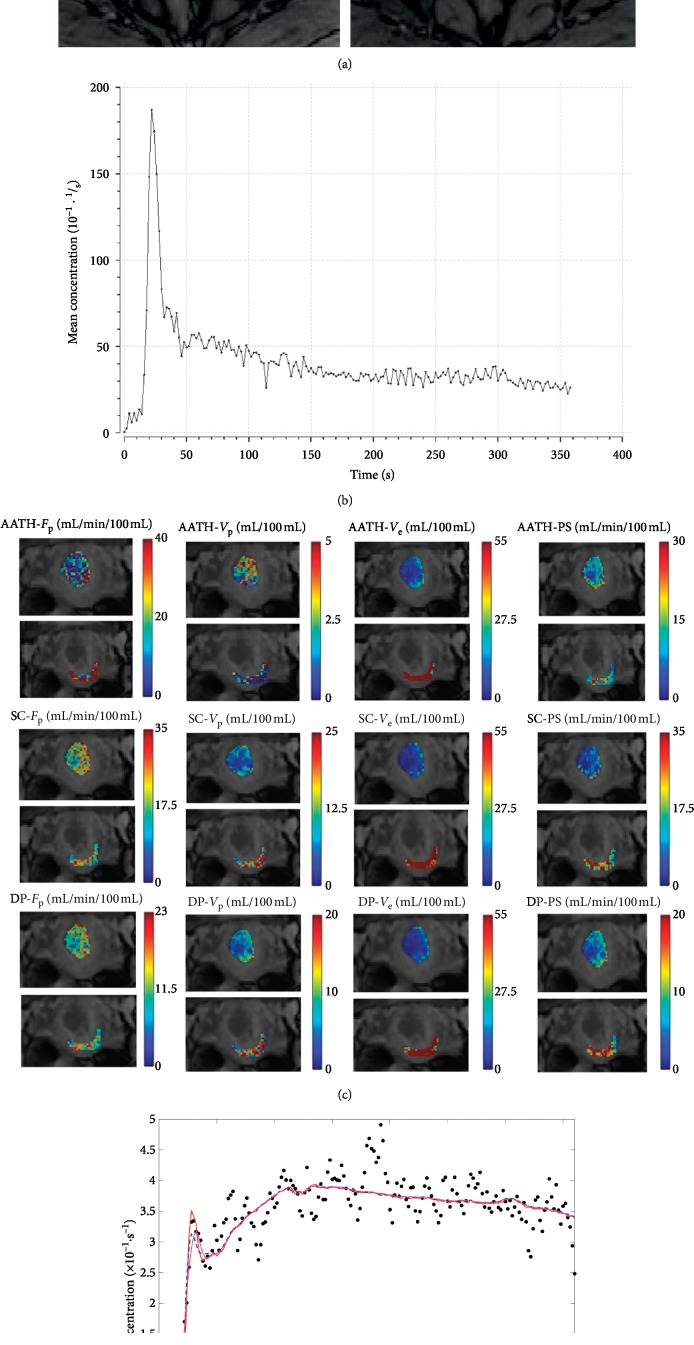
Example of a patient with stage IIb cervix cancer. (a) ROIs for cervix carcinoma (blue) and normal cervix tissue (red) are shown for the central four slices of the DCE-MRI dataset, and the location within the iliac artery where the AIF was sampled was marked with a red dot. (b) Sampled AIF used in model fitting. (c) Parameter maps generated using the three models (AATH, SC, and DP) for tumor and the normal tissue ROIs. (d) Examples of curve fittings for a tumor voxel. In the legend, the four numbers within square brackets beside each model are their respective parameter values: (*F*_p_ (mL/min/100 mL), *V*_p_ (mL/100 mL), PS (mL/min/100 mL), *V*_e_ (mL/100 mL)).

To avoid possible effects of inflow and inhomogeneity near boundaries, only the central four slices from the imaging volume (of 6 slices) were selected for processing. For each patient, regions of interest (ROIs) for tumor lesions and normal cervix tissue were manually delineated on the central four slices (as illustrated in Figure 1(a)) by two experienced radiologists with more than 10 years of experience in gynecological radiology. Routine T1-weighted, T2-weighted, and DW images were used for cross-referencing to confirm the location and size of the lesions when contrast-enhanced scans were evaluated. The size of ROI is no less than 10 voxels to ensure the robustness of measurement. The normal ROIs were selected in the normal cervical tissue away from the lesions. The areas of necrotic, cystic, and hemorrhages were avoided when drawing the lesion ROIs. Finally, 107 ROIs for cervix cancer were obtained from 36 patients. 103 ROIs for normal tissues were obtained from 17 healthy subjects and 24 patients with smaller masses which can be delineated accurately. AIF was sampled from a voxel that clearly resided within the iliac artery on one of the four central slices as shown in Figure 1(b). Desirable features for AIF selection included an early bolus arrival time and high peak value and signal-to-noise ratio. Fitting of voxel-level tissue concentration-time curves *C*_tiss_(*t*) and generation of parametric maps were performed using a commercially available software (MItalytics, Fitpu Healthcare, Singapore). The software allows for the selection of an individual AIF for each patient case and employs a constrained nonlinear optimization algorithm in fitting the various models.

### 2.5. Statistical Analysis

For each patient, the median parameter value of all voxels within the tumor ROIs on multiple slices is taken as a representative statistic of the parameter in the tumor. The median values of the fitted parameters were used because the median is more robust to outliers (that could occur during data fitting) than the mean. A two-way model average measure, intraclass correlation coefficient (ICC), was used to test the interobserver consistency. Then, all the measurements from the two observers were averaged for further comparison. Agreement was interpreted according to the ICC as follows: >0.80, excellent; 0.61–0.80, good; 0.41–0.60, moderate; 0.21–0.40, fair; and <0.2, poor agreement [27].

The normality of the distribution of all parameters was analysed by the Kolmogorov–Smirnov test. The receiver operating characteristic (ROC) curves of all parameters were obtained and the areas under the curves (AUC) were evaluated to determine the discriminating power of DCE parameters between the lesion and normal tissues. Interpretation of AUC values is application dependent, and in general, it is appropriate that values ≥0.9 would be “excellent,” ≥0.8 “good,” ≥0.7 “fair,” and <0.7 “poor” [28].

The Pearson correlation coefficient *r* was used to explore possible relationship between median parameter values of the three models. A strong correlation was assumed for 0.8 < *r* ≤ 1, a moderate correlation for 0.5 < *r* ≤ 0.8, a weak correlation for 0.3 < *r* ≤ 0.5, and no correlation for *r* ≤ 0.3 [29]. The Bland–Altman plot was used to show agreements between parameter values of the three models. All statistical analysis were performed using SPSS software 18.0 (Chicago, IL, USA), and *P* < 0.05 was considered statistically significant.

## 3. Results

For the case shown in Figure 1(a), the parameter maps of one slice generated using the three methods (AATH, SC, and DP) are shown in Figure 1(c). It is evident that three methods attain similar results in *V*_e_ and PS, and both estimates are smaller in tumor ROI than in normal tissue ROI. For *F*_p_, the estimate by AATH is smaller in tumor ROI and the estimates by SC and DP are apparently close between tumor and normal tissue ROI. For *V*_p_, the estimates vary from close between tumor and normal tissue ROI (AATH) to smaller in tumor ROI (SC and DP).

Table 2 shows the ICC values for the measured parameters, where most ICC values are greater than 0.9, indicating very good agreement between measurements from two observers. Thus, the parameter values as measured by two observers are averaged and utilized in the analysis as follows.

Table 3 shows the nonparametric statistical analysis of parameters between cervix cancer and normal tissue ROIs. The parameters *F*_p_, *V*_e,_ and PS of the three DCE models (AATH, SC, and DP models) were smaller in cervix cancer lesions than in normal cervix tissue, and the differences between lesions and normal tissue were mostly significant except for PS from the AATH model and *F*_p_ from the SC model. *V*_p_ by AATH and SC was significantly larger in cervix cancer lesions than in normal cervix tissue, and DP yielded contradictory results, though the difference between lesions and normal tissue was not statistically significant. Parameter *V*_e_ attained fair to good AUC values (>0.83 for AATH and DP models and >0.75 for the SC model). Other parameters, e.g., *V*_p_ from the AATH model and PS from the SC model also showed fair AUC values.

**Figure 2:**
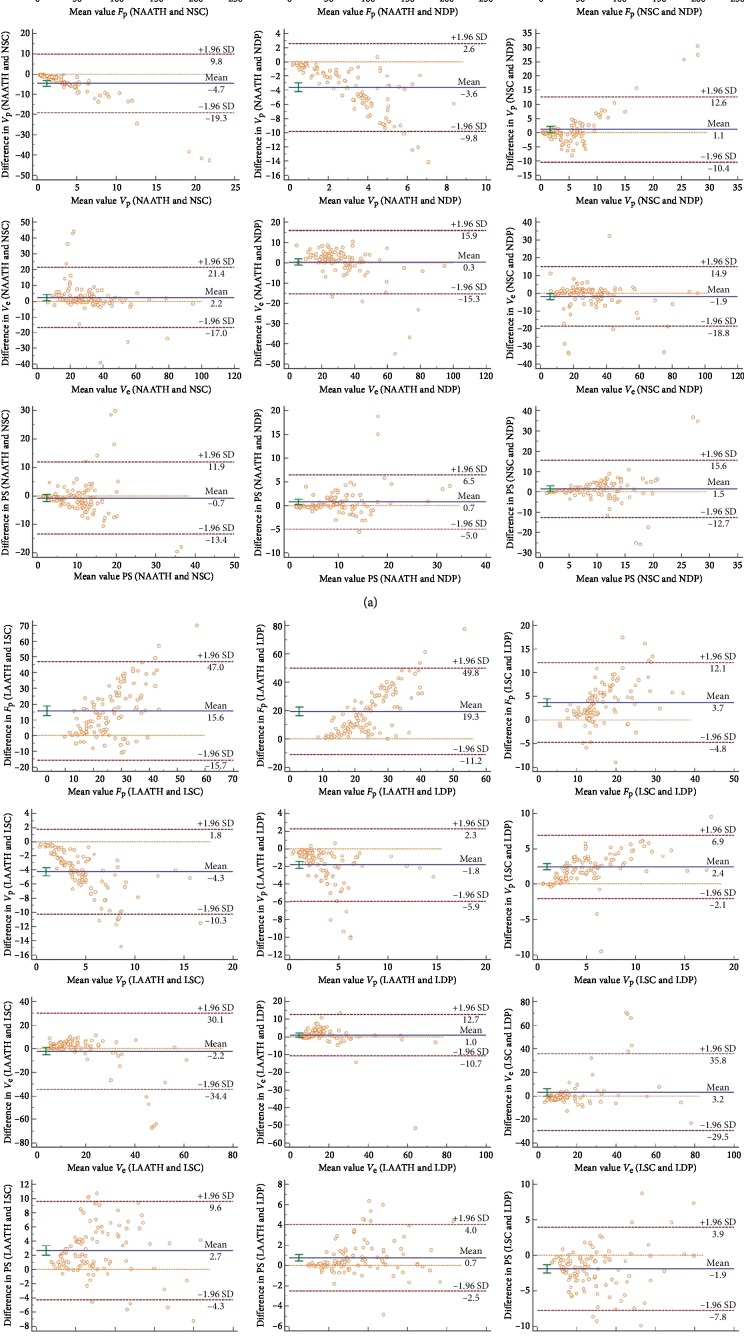
(a) Bland–Altman plots for *F*_p_, *V*_p_, *V*_e,_ and PS from AATH, SC, and DP models in normal cervix tissue (N). (b) Bland–Altman plots for *F*_p_, *V*_p_, *V*_e,_ and PS from AATH, SC, and DP models in the cervix lesion (L). The unit of these parameters is listed as follows: *F*_p_ (mL/min/100 mL), *V*_p_ (mL/100 mL), *V*_e_ (mL/100 mL), and PS (mL/min/100 mL).

Results of the Pearson correlation between the same parameters estimated by different models are shown in Table 4. Comparing parameter estimates of the three models (AATH, SC, and DP models) for cervix cancer lesions, it was found that *F*_p_ and *V*_p_ from SC and DP models and *V*_e_ and PS from AATH and DP models were highly correlated, respectively (*r* > 0.8). Moderate correlations were observed between *V*_p_ from AATH and *V*_p_ from SC and DP models, *V*_e_ and PS from AATH and SC models, and *V*_e_ and PS from SC and DP models (0.5 <*r* <0.8). No correlation was observed between *F*_p_ from AATH and *F*_p_ from SC and DP models, respectively (*r* *<* 0.3). Comparing parameter estimates of the three models (AATH, SC, and DP models) for normal cervix tissues, it was found that good agreement existed between the estimates of *F*_p_, *V*_e_ from the three models, and PS from AATH and DP models (*r* > 0.8). Moderate correlations were observed between PS from AATH and SC models and *V*_p_ from SC and DP models (0.5 < *r* < 0.8). A weak correlation was observed between PS from SC and DP models (0.3 < *r* < 0.5). No correlation was observed between *V*_p_ from AATH and *V*_p_ from SC and DP models, respectively (*r* *<* 0.3). Results of the Bland and Altman test for comparison of the parameter with the same biophysical meaning obtained using the three models are shown in Figure 2. Bland–Altman plots demonstrated good agreements between the estimates of *V*_e_ from the three models for both normal cervix tissue and cervix lesion because their *y*-coordinate values were centered at zero (Figures 2(a) and 2(b)). Good agreements were observed between *V*_p_ from SC and DP models and PS from AATH and SC models in normal cervix tissue (Figure 2(a)). The *y*-coordinate value of other parameters estimated by AATH, SC, and DP modes stayed away from zero, indicating less agreement between them in normal cervix tissue and cervix lesion.

## 4. Discussion

This preliminary study evaluated the performance of physiological parameters derived from AATH, SC, and DP models with respect to tumor microcirculation in cervix cancer. The angiogenic activity of cervix cancer assessed by various DCE-MRI models improved *in vivo* understanding of the fundamental processes involved in tumor angiogenesis. Parameter *V*_e_ has the best performance in identifying cervix cancer among all parameters. Most physiological parameters derived from the three DCE-MRI models are linearly correlated in cervix cancer.

The parameter *V*_e_ of all three DCE models showed that the *V*_e_ value of cervix cancer tissue was significantly smaller than that of normal cervix tissue. The corresponding AUC values were greater than 0.75, and *V*_e_ of the AATH model had the largest AUC (0.85) among all parameters, indicating a fairly good performance in terms of diagnostic value. In tracer kinetic modeling, *V*_e_ stands for the fractional volume of extravascular extracellular space, which is closely pertaining to the degree of cell growth. The more the cells grow, the less the *V*_e_ value is. Tumor is typically characterized by the over-growth of cells. Thus, the observed smaller value of *V*_e_ in cervix cancer tissue suggests the over-growth of cells in cervix cancer tissue in comparison with normal cervix tissue, which is consistent with the feature of other cancer tissue.

In the tissue region of interest, there are three types of transportation process for the tracer molecule: the flowing process within the vascular space, the exchange process between the vascular and extravascular extracellular space, and the diffusion process within the extravascular extracellular space. The first two processes are of interest in DCE tracer kinetic modeling, and the associated parameters are *F*_p_ for the flowing process and PS for the exchange process, both of which are linked to nutrition supply to cell growth. In most solid tumors, both parameters usually increase due to the over-growth of tumor cells. From Table 2, it is observed that both parameters as estimated by the three models are surprisingly smaller in cervix cancer tissue.

Another parameter in DCE modeling, which is also closely relevant to cell growth, is *V*_p_, the fractional volume of vascular space, characterizing the degree of microvascularity in tissue. In general, tumor tissue would develop more microvessels as a response to nutrition necessity in tumor cell over-growth. Parameter *V*_p_ can be validated through pathohistological experiment, which can visualize and quantify the degree of microvascularity through the measure of microvessel density (MVD). Early experiments showed that the MVD of cervix cancer tissue was larger than that of normal cervix tissue [30–32]. In this study, it is observed that AATH and CC models yielded *V*_p_ values consistent with previous pathohistological results, and the DP measurement is on the contrary, but with insignificant difference in value.

**Figure 3:**
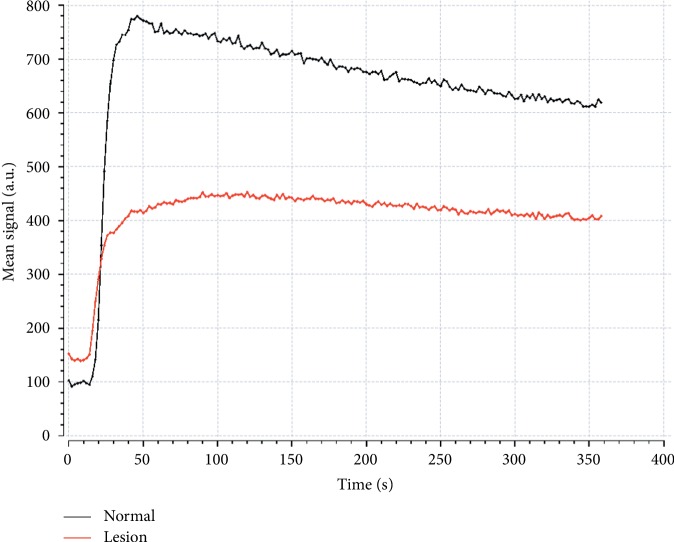
The signal intensity-time curve of cervix cancer ROI and the normal tissue for the same patient in Figure 1.

The unusual observation in the microcirculation pattern of cervix cancer tissue is also evidently reflected in Figure 3, which exemplifies the change of tissue intensity after venous injection of contrast media. The baseline corresponds to the period the contrast media has not flowed into the tissue of interest. Here, the intensity of cervix cancer tissue is slightly brighter than that of normal cervix tissue, indicating that cervix cancer tissue has shorter spin-lattice relaxation time. With the arrival of contrast media, tissue intensity rapidly increases till a “peak” value, followed by a part with intensity slowly decreasing, which stands for the wash-in phase and wash-out phase of contrast media, respectively. The speed of uptake in the wash-in phase depends on the value of blood flow, and the larger the value, the sharper the slope of the uptake curve. The sharper slope of normal cervix tissue suggests a lower blood flow for the cervix cancer tissue, which is well in line with the observation in DCE modeling. The “peak” value is primarily related to the degree of microvascularity. If the tissue develops more microvessels, there would accumulate more tracer molecules, hence to observe brighter intensity value. The lower “peak” value of cervix cancer tissue suggests that cervix cancer tissue might develop fewer vessels. This is consistent with the finding by the DP model, but not consistent with either that by AATH and CC models or previous pathohistological experiment. This apparent contradiction might be due to the dysfunction of vessels newly developed in cancer tissue [33], which may accumulate less tracer molecules, resulting in the lower “peak” value of cervix cancer.

The wash-out phase involves two processes: one process where the contrast media flows out of the tissue of interest and another process where the contrast media leaks out of vascular space through vascular wall and enters the extravascular extracellular space and returns from the extravascular extracellular space to the vascular space and flows out of the tissue of interest. The flow-out process would lead to lower tissue intensity, and the exchange process would determine the speed of intensity decreasing. The higher the blood flow is, the faster the intensity decreases. The more leakier the vessel wall is, the slower the intensity decreases. The trend of wash-out phase would be determined by both factors. If the blood flow is large and the effect of flow-out process is dominant, a downward trend would be observed. If the blood flow is small and the vessel wall is leaky, a slowly upward or flat trend could be observed. As there is an evident downward trend in the wash-out phase of normal cervix tissue, it demonstrates the higher blood flow rate in normal cervix tissue. Consequently, the intensity difference between cervix cancer tissue and normal cervix tissue is decreasing in the early stage of wash-out phase. In spite of this, it is clear that the intensity difference becomes stable towards the tail stage of the observed wash-out phase, which is more influenced by the exchange process. As aforementioned, the measured permeability PS in DCE modeling is larger in normal cervix tissue; thus, the downward trend in normal cervix tissue encounters larger resistance, which helps precisely to explain the observed phenomena in the wash-out phase.

From the aforementioned analysis, cervix cancer exhibits very unusual pattern in microcirculation, with over-growth of cells, decreased blood flow, decreased vessel wall permeability, and without well development of increased and functional microvascularity. One immediately notices what is the growing mechanism for tumor cells without additional nutrition supply through the blood transportation system. It is known that microvascular proliferation is not the only factor of cancer cell growth and some other factors like interstitial fluid pressure, oxygen, and human papilloma virus (HPV) [9, 34, 35] but may also promote tumor growth. But, which of these pathophysiological mechanisms is the dominating factor for the growth of cervix cancer cells is not clear so far. It would require a histopathologic and etiologic study to uncover the difference of growth condition between cervix carcinoma and other tumors. For clinicians to provide individual cancer patients with optimal treatment and precise prognosis, further knowledge on the tumor microenvironment would be of great value.

In the situation where two different kinetic models were employed in a multicenter clinical trial for the purposes of assessment and monitoring of treatment effect using DCE-MRI, it would be desirable that the kinetic parameters estimated by the two models can be related, i.e., that they were at least linear correlated. Parameter *V*_e_ derived from all three models, *V*_p_ estimated by SC and DP models, and PS from AATH and SC models in cervix lesion were highly correlated as well as in good agreement, indicating a fairly good performance in terms of evaluation of treatment effect in multicenters. In most situations, parameters derived from different models were not quantitatively equivalent but highly correlated. For example, if the SC and DP models were employed in different centers and one center observed a decrease in perfusion (tissue blood flow) of cervix cancer estimated by SC-*F*_p_ due to a particular treatment, then another center employing the DP model should also observe a similar percentage decrease in DP-*F*_p_. Because SC-*F*_p_ and DP-*F*_p_ were highly correlated but not quantitatively equivalent, we could only compare their relative difference (percentage change) in *F*_p_ before and after treatment and not their absolute values. In a DCE CT study of brain tumors [36], parameters *F*_p_, *V*_p_, *V*_e_, and PS from the SC model were found to be highly correlated with those from the DP model. These correlation results for these parameters in meningiomas derived from DCE CT are very similar to the present results derived from DCE-MRI for cervix cancer (*r* > 0.5). However, comparing parameter estimates of the three models (AATH, SC, and DP models) for cervix in this study, it was found that the Pearson correlation between parameters of the three models in cervix cancer lesion and normal cervix tissue yield inconsistent conclusion, i.e., *F*_p_ from AATH model and *F*_p_ from SC and DP models were highly correlated, respectively, in normal cervix tissue, but very weak correlation in cervix cancer lesions. *V*_p_ from the AATH model and *V*_p_ from SC and DP models were highly correlated, respectively, in cervix cancer lesions, but very weak correlation in normal cervix tissue. (Table 4). Thus, the parameter correlations between kinetic models of tumors may be different from normal tissue, and it should not be generalized without validation for different tumors characterized with different perfusion. Parameter relationships under various tracer kinetic models in other tumors need further investigation.

This study has a few limitations. There was a lack of pathohistological experiment, and the previous pathohistological results by other researchers were employed for comparison in this study. The present DCE-MRI data were acquired using a protocol with high temporal resolution (2 s) in order to capture rapid changes in arterial and tumor concentration with time, which led to a tradeoff in organ coverage and image spatial resolution. As a result, effects of lesion heterogeneity might not be fully accounted for. Possible error sources that could affect contrast concentration estimation in this study include the lack of correction for effects of (i) B1 field inhomogeneity and (ii) T2*∗* relaxation. (i) Concentration estimation based on variable flip angle T1 mapping would be sensitive to spatial nonuniformity in the radio-frequency transmit field (B1) which causes variations in the prescribed flip angles. (ii) At high-contrast concentrations, effects of T2*∗* relaxation become increasingly substantial in higher field strengths and could not be ignored during concentration estimation. Previous studies have shown that failure to account for T2*∗* effects could result in underestimation of the AIF, especially for concentrations around the AIF peak [37, 38]. Both effects (i) and (ii) can result in errors in the arterial and/or tissue concentration-time curves, which further translate to errors in the estimation of kinetic parameters [37, 38]. A major limitation of this study is therefore the lack of quantification of such errors and their propagation to the estimated kinetic parameters, which would be the subject of further study in our future work.

In conclusion, two-compartment exchange models turned out to be very promising tools in analyzing DCE data and assessing the microcirculation pattern in cervix cancer tissue, hence with great value to assist cervix cancer diagnosis and prognosis. Parameter *V*_e_ has the best performance in identifying cervix cancer tissue among all parameters.

## Figures and Tables

**Table 1:** Patient characteristics.

Parameters	No.of patients
Age; mean (range) years	42 (42–75)
Histological subtype	
Adenocarcinoma (AC)	2
Squamous cell carcinoma (SCC)	34
Tumor grade	
Well	6
Moderate	30
FIGO^a^ stage	
Ib	17
IIa	14
IIb	5

Abbreviation: FIGO International Federation of Gynecology and Obstetrics. ^a^According to FIGO 2009 staging criteria.

**Table 2:** Interobserver consistency for cervix lesion and the normal cervix tissue.

	AATH				SC				DP			
	*F*_p_	*V*_p_	*V*_e_	PS	*F*_p_	*V*_p_	*V*_e_	PS	*F*_p_	*V*_p_	*V*_e_	PS
Lesion												
ICC	0.922	0.975	0.959	0.939	0.935	0.980	0.968	0.857	0.965	0.989	0.958	0.958
95% CI	0.886–0.947	0.963–0.983	0.940–0.972	0.911–0.958	0.905–0.956	0.971–0.987	0.953–0.978	0.790–0.903	0.949–0.976	0.984–0.993	0.938–0.971	0.938–0.971
Normal												
ICC	0.934	0.661	0.908	0.948	0.978	0.945	0.910	0.887	0.906	0.812	0.943	0.892
95% CI	0.902–0.955	0.499–0.770	0.864–0.938	0.924–0.965	0.967–0.985	0.919–0.963	0.867–0.939	0.833–0.923	0.861–0.936	0.702–0.882	0.916–0.961	0.841–0.927

ICC, intraclass correlation coefficient. CI, confidence interval of difference.

**Table 3:** Comparison of model parameters between cervix carcinoma and normal cervix tissue.

	*F*_p_ (mL/min/100 mL)	*V*_p_ (mL/100 mL)	*V*_e_ (mL/100 mL)	PS (mL/min/100 mL)
AATH-normal cervix	44.57 ± 23.23	1.27 ± 1.25	32.15 ± 14.32	10.40 ± 6.43
AATH- cervix cancer	34.13 ± 15.15	2.55 ± 2.30	17.03 ± 10.80	8.88 ± 4.28
*P*	<**0.001** ^a^	<**0.001** ^a^	<**0.05** ^a^	>0.05^a^
AUC	0.67	0.75	0.85	0.56

SC-normal cervix	19.51 ± 15.96	6.00 ± 7.65	29.95 ± 17.33	11.14 ± 7.18
SC-cervix cancer	18.48 ± 7.31	6.80 ± 4.24	19.21 ± 20.05	6.21 ± 4.40
*P*	>0.05^b^	<**0.001** ^a^	<**0.001** ^a^	<**0.001** ^a^
AUC	0.50	0.64	0.75	0.75

DP-normal cervix	16.06 ± 21.22	4.90 ± 3.24	31.88 ± 18.41	9.67 ± 5.62
DP-cervix cancer	14.83 ± 5.23	4.38 ± 3.06	16.05 ± 13.34	8.13 ± 3.96
*P*	<**0.01** ^a^	>0.05^b^	<**0.001** ^a^	<**0.05** ^a^
AUC	0.60	0.54	0.83	0.58

AUC, area under the ROC curve. ^a^Comparison was performed by the Mann–Whitney *U* test. ^b^Comparison was performed by the independent *t* test.

**Table 4:** Results of the Pearson correlation between parameters of the three models (AATH, SC, and DP) in cervix cancer carcinoma and normal cervix tissue. *r* > 0.5 and *P* < 0.05 are indicated by^*∗*^.

	*F*_p_ (mL/min/100 mL)	*V*_p_ (mL/100 mL)	*V*_e_ (mL/100 mL)	PS (mL/min/100 mL)
Lesion				
AATH-CC	0.121 (*P*=0.214)	0.710 (*P* < 0.001)^*∗*^	0.572 (*P* < 0.001)^*∗*^	0.667 (*P* < 0.001)^*∗*^
AATH-DP	0.095 (*P*=0.329)	0.732 (*P* < 0.001)^*∗*^	0.899 (*P* < 0.001)^*∗*^	0.921 (*P* < 0.001)^*∗*^
CC-DP	0.813 (*P* < 0.001)^*∗*^	0.850 (*P* < 0.001)^*∗*^	0.565 (*P* < 0.001)^*∗*^	0.749 (*P* < 0.001)^*∗*^

Normal				
AATH-CC	0.829 (*P* < 0.001)^*∗*^	0.261 (*P*=0.008)	0.824 (*P* < 0.001)^*∗*^	0.554 (*P* < 0.001)^*∗*^
AATH-DP	0.815 (*P* < 0.001)^*∗*^	0.247 (*P*=0.012)	0.912 (*P* < 0.001)^*∗*^	0.890 (*P* < 0.001)^*∗*^
CC-DP	0.981 (*P* < 0.001)^*∗*^	0.696 (*P* < 0.001)^*∗*^	0.886 (*P* < 0.001)^*∗*^	0.385 (*P* < 0.001)

## Data Availability

The data of this study are already presented in this paper.
